# Toward a Contextually Sensitive Understanding of Polyvictimization: A Latent Class Analysis of Violence, Risks, and Protections Among South African Adolescents From Highly Deprived Settings

**DOI:** 10.1177/08862605241233273

**Published:** 2024-02-26

**Authors:** Hannabeth Franchino-Olsen, Mark Orkin, Franziska Meinck

**Affiliations:** 1 University of Edinburgh, UK; 2 University of the Witwatersrand, Johannesburg, Gauteng, South Africa; 3 North-West University, Vanderbijlpark, South Africa

**Keywords:** polyvictimization, violence, abuse, adolescent, HIV, South Africa

## Abstract

South African adolescents experience a high prevalence of violence victimization alongside the health and economic burdens of HIV/AIDS and poverty. Polyvictimization is a useful theory and framework that allows for a nuanced understanding of lived adolescent experience patterns. Polyvictimization examinations are further enriched by person-centered analytical approaches. This study used latent class analysis to differentiate a sample of South African adolescents from highly deprived communities by their polyvictimization profiles and contextual violence risk and protective factors. Adolescents were sampled twice (2010/2011; 2011/2012), and data reflected their lifetime (sexual abuse) or recent (all other forms of assessed abuse/violence) violence victimizations, as well as individual, household, and community characteristics. Model fit indices supported a seven-class model with adolescents in high, moderate, and low polyvictimization classes. Adolescents in the high polyvictimization classes experienced a heavy burden of poverty and multiple forms of violence across contexts and were distinguished by HIV/AIDS and disability. Adolescents in the low polyvictimization class experienced relatively little violence, despite living in violent communities, and low household and individual burdens of HIV/AIDS and disability. Findings emphasize the importance of considering adolescent violence through a contextually sensitive polyvictimization lens to understand the complex web of violence that adolescents experience. This work supports previous research in low-resource South African settings highlighting the interconnected nature of violence, poverty, disability, and HIV/AIDS. Future research should explore these complex violence patterns and their effects, while program and policy actions must target and prevent adolescent violence especially for those impacted by poverty, disability, and HIV/AIDS.

## Background

Violence experienced by adolescents is a relevant public health concern, as violence victimization before adulthood is linked to numerous poor mental and physical health outcomes in adolescence and across the life course (Kessler et al., 2010; Leeb et al., 2011; Norman et al., 2012). Understanding and eradicating violence is especially important for children in highly deprived, low-resource communities in South Africa, where overlapping burdens and epidemics of poverty, HIV/AIDS, family and community violence, and limited educational supports place undue risks on their health and well-being from birth through adolescence (Panday et al., 2013; Ward et al., 2015). In South Africa, previous work has demonstrated the high prevalence of violence and abuse experienced by adolescents in these contexts (Meinck et al., 2016). Frequent violence there has been linked to educational delays, mental health difficulties, and poor health behaviors (Cluver et al., 2018; Herrero Romero et al., 2019; Humm et al., 2018).

Evidence from a variety of contexts indicates that young people (adolescents and young children) often experience more than one form of violence before entering adulthood (Alexander et al., 2021; Finkelhor et al., 2007a; Hamby & Grych, 2013; Turner et al., 2016). Young people may experience multiple forms or types of violence in their homes, schools, and/or communities in young childhood and adolescence (Butcher et al., 2016; Hamby & Grych, 2013). This seems to be the case in specific South African contexts, where previous exposure to violence and/or living in a high-violence community increased the risk of subsequent violence victimizations, such as bullying or sexual abuse (Cluver et al., 2010; Meinck et al., 2015b). Given this evidence, the polyvictimization theory and framework developed by Finkelhor et al. (2011) is salient in its recognition of multiple victimization events and forms across and within contexts, allowing for the broad inclusion of violence occurring in families, schools, peer groups, romantic partnerships, and communities (Willie et al., 2017). Though it is defined and operationalized in various ways by researchers (Song et al., 2022; Willie et al., 2017), this study defines polyvictimization to mean experiencing multiple forms of violence, such as multiple forms of abuse, community violence, domestic violence, and/or peer violence (Dierkhising et al., 2019; Lasky et al., 2021). Polyvictimization acts as a framework in which a diverse constellation of violence experiences can be examined and contextualized, which is a particularly powerful tool when examining a period in the life course characterized by major transitions, such as adolescence (Hamby & Grych, 2013).

Polyvictimization has been successfully studied among diverse adolescent samples, though most of the global research on this topic has been conducted in samples from high-income countries, particularly the United States and Europe (Butcher et al., 2016; Charak et al., 2019; Finkelhor et al., 2007b; Ford et al., 2013; Kretschmar et al., 2017; Le et al., 2018; Sui et al., 2021). Though less examined in low-income contexts, a systematic review by Le et al. (2018) regarding polyvictimization in low- and lower-middle-income countries (LALMIC) found that in these poorer settings adolescent polyvictimization was more common than in high-income contexts. In their review, sub-Saharan African countries—Democratic Republic of the Congo, Kenya, Sierra Leon, Tanzania, and Uganda—were represented in thirteen studies. Adolescent polyvictimization prevalence, when reported, ranged from 47% to 63% (Le et al., 2018). Though South African samples were not represented in this LALMIC review, individual studies with South African adolescents found that they experienced a markedly high prevalence of violence in many contexts (Sui et al., 2021). In one sample from adolescents living in Cape Town, 40% had experienced community violence, 77% had witnessed domestic violence, 59% had been victimized or abused at home, 76% had experienced violence at school, and 8% had been sexually abused; violence in each of these contexts was found to be highly correlated with 93% of the sample experiencing more than one form of violence and 55% experiencing four or more forms of violence (Kaminer et al., 2013). This high prevalence of polyvictimization was again shown in a population-based sample of South African adolescents, which found that only 36% experienced no or single victimization, while the rest of the sample had experienced low (29%), high (23%), or very high (13%) polyvictimization (Leoschut & Kafaar, 2017). Examination of nationally representative data from South African adolescents supported the interconnected nature of violence/abuse experiences inherent in polyvictimization with adolescents who experienced sexual violence having increased odds of also experiencing other direct and indirect victimizations, including physical abuse, emotional abuse, neglect, family violence (Ward et al., 2018). These South African findings align with the findings in other low- and middle-income settings and even high-income settings: for adolescents, violence is frequently spread across multiple forms and contexts (Kaminer et al., 2013; Le et al., 2018; Leoschut & Kafaar, 2017).

Conceptualizing adolescent interpersonal violence experiences with a polyvictimization lens allows consideration of the interconnected nature of violent events—or web of violence—in the lives of adolescents, rather than fixating on or overemphasizing the importance of a single form of adolescent violence (Butcher et al., 2016; Davis et al., 2019). Polyvictimization theory posits that individuals who experience multiple forms of violence victimization are likely to experience more severe or longer detrimental impacts to their health and well-being than those who experience only a single form of violence (Alexander et al., 2021; Butcher et al., 2016; Davis et al., 2019; Finkelhor et al., 2007a, 2007b). The theory also emphasizes the need to consider risk factors for health and violence beyond the victimization experiences alone in order to broaden the context and associated factors occurring with the violence in an individual’s life (Butcher et al., 2016; Davis et al., 2019). For example, adolescents living in resource-poor areas of South Africa may carry or experience multiple risk factors that increase their risk of violence or magnify the harm of violent experiences. These include living in poverty, providing care for an AIDS-sick family member, experiencing HIV-related stigma, having a disability, or engaging in alcohol or drug use (Meinck, Cluver, Boyes et al., 2015; Skeen et al., 2015).

The consideration of multiple forms of violence alongside additional risk factors and social and/or demographic factors can create enormous complexity when investigating polyvictimization via the many potential combinations of violence experiences and risk factors. This has previously been addressed in the literature through the use of mixture modeling—including latent class analysis and growth mixture modeling—which have allowed researchers to examine heterogeneous victimization experiences inherent in polyvictimization by analyses which allow individuals to be grouped by shared or common violence experiences (Butcher et al., 2016; Davis et al., 2019). Person-centered methods, such as latent class analysis, allow for a nuanced and sensitive investigation into patterns or subgroups in a sample, which is a valuable investigational technique when considering diverse violence patterns via polyvictimization (Davis et al., 2019). Latent class analysis provides an empirically based approach to teasing out polyvictimization in a sample and is a meaningful tool for policy and programming response, as it indicates the size (*n*) of each generated class to allow targeted allocation of resources according relative class sizes (Willie et al., 2017). Additional variables beyond those relevant to violence and polyvictimization can also be included in latent class models, allowing the creation of classes that account for contextually specific factors (Mathur et al., 2020).

The current study has extended this precedent by using latent class analysis to examine the associated risk and protective factors as well as the polyvictimization experiences among a sample of Black South African adolescents from low-resource, highly deprived communities with a high burden of HIV/AIDS. To our knowledge, this is a unique addition to the literature as no previous work has examined polyvictimization among South African adolescents using a person-centered analytical perspective among a sample that includes highly deprived and rural settings and in which HIV/AIDS prevalence is notably high. The inclusion of relevant contextual factors alongside violence experiences is key to this analysis as it considers the stressors, burdens, and social phenomena, such as poverty and HIV/AIDS, which act as drivers for violence and increase the risk of harm from violence experiences, including polyvictimization (Brown et al., 2023). The research questions for this analysis accordingly were: (a) Using latent class analysis with inclusion of relevant risk and protective factors, how do forms of adolescent violence victimizations—domestic violence exposure, community violence, child abuse, and bullying—group together for adolescents from these low-resource South African communities and what polyvictimization profiles are created?; and (b) What are the associated risk factors—including indicators for community setting, poverty, household, and caregiver characteristics—and protective factors—government grants and schooling benefits—for polyvictimization latent class groupings?

## Methods

### Sample

The study analyzed data from the longitudinal Young Carers project. Adolescents were recruited at wave 1 from randomly selected census enumeration areas within two South African provinces (Western Cape and Mpumalanga) in 2010 to 2011. Door-to-door sampling identified households with eligible adolescents (aged 10–17) and less than 2.5% of eligible adolescents refused to participate at wave 1 (*n*_wave1_ = 3,515). Wave 2, which was completed approximately one year later in 2011 to 2012, included 3,401 of the original respondents who were aged 12 to 18 in this follow-up interview.

At each wave, adolescents were interviewed by trained local interviewers in their home language (XiTsonga, SiSwati, SiPedi, or isiXhosa) using a questionnaire with validated scales which asked about violence exposure, risk behaviors, poverty, family health and relationships, mental health, communities, and social protections received. More information on the sample and collected data including eligibility, recruitment, consent procedures, and interview measures can be found elsewhere (Meinck et al., 2016; Meinck, Cluver, Loening-Voysey et al., 2017; Supplemental Appendix 1). Given the research questions’ focus on polyvictimization, this study examined data from participants included in both wave 1 and wave 2 samples. Composite variables representing violence victimization experiences reported at wave 1 and/or wave 2 were created to capture all experiences of violence examined by the questionnaires. Other than sexual abuse (which was assessed both for recent experiences (wave 2) and across the participant’s lifetime [wave 1, wave 2]), recent experiences of violence were assessed at each wave (events in the last month to the last year). To capture the diversity of violent experiences and shifting life events available in our adolescent sample, the violence measures and nondemographic risk and protective factors drawn from the sample reflected a composite of experiences that occurred at wave 1, wave 2, or both waves. Thus, this sample, though drawn from a longitudinal study design, is cross-sectional in nature to provide insight into the constellation of violence victimizations reported by adolescents and to allow this work to examine violence which occurred prior to recruitment in the study (wave 1 questionnaire) and between wave 1 and wave 2 interviews (wave 2 questionnaire).

### Measures

#### Violence Measures

Adolescent participants’ experiences of household, family, peer, and community violence were assessed in both interviews at waves 1 and 2. Specific information about the scales used for each violence measure can be found in Supplemental Appendix 1. Measures used standardized scales, were previously shown to reliable in South Africa and all demonstrated to be reasonably reliable in this sample (α > .8). For domestic violence exposure (drawn the UNICEF Measures for National-level monitoring of orphans and other vulnerable children; Snider & Dawes, 2006), participants reported whether adults in their house engaged in arguments with shouting or arguments with hitting. Using the Child Exposure to Community Violence Checklist (Richters & Martinez, 2004), participants who reported being robbed or physically assaulted in their community were considered to have experienced community violence victimization, while those who witnessed someone in their community be shot or stabbed were coded as having witnessed community violence.

Physical and emotional items came from the same UNICEF Measures used for domestic violence exposure (Snider & Dawes, 2006) and sexual abuse items came from the Juvenile Victimization Questionnaire (Finkelhor et al., 2005). Physical abuse items assessed whether the participant had been hit with an object by their caregiver or hurt when slapped, punched, or hit by their caregiver. Emotional abuse items asked if they had been kicked out of their home, threatened with ghosts, evil spirits, or harmful people, or called dumb, lazy, or other names by their caregiver. Sexual abuse items assessed nonconsensual contact sexual activity, as well as sexual assaults and attempted sexual assaults. Participants who reported any of these experiences were considered to have experienced physical abuse, emotional abuse, or sexual abuse, respectively.

Bullying items (drawn from the Social and Health Assessment Peer Victimization Scale; Ruchkin et al., 2004) asked about experiences in the last year and included questions about verbal and physical events, intimidation, and social isolation. Most participants (91.6%) reported at least one of the bullying items as occurring once or more in the last year. To capture participants who experienced frequent recurrence of bullying events or a diversity of bullying victimization events, participants were coded as experiencing bullying if they reported three or more of the bullying items as occurring two to three times or at least one of the bullying items as occurring four or more times. All violence measures were coded as binary for the latent class analyses (LCA).

#### Demographic and Household Measures

The setting in which the participant lived at wave 2 was coded dichotomously to reflect rural versus urban communities. Household poverty was captured via measures of reported hunger by the participant (if they went to bed hungry in past week or were hungry at school; via the South African National Food Consumption Survey; Labadarios et al., 2003) and a measure of necessities affordable for the household (via Indicators of Poverty and Social Exclusion Project; Barnes & Wright, 2012; Pillay et al., 2006). Participants were coded as experiencing hunger if they reported recent experiences of hunger at home or school and were coded as lacking necessities if they were unable to afford four or more necessities from the list of eight necessary household expenses (e.g., three meals per day, toiletries, clothes, and medical care). Housing security was captured by assessing whether the participant’s dwelling qualified as informal housing using measures from the South African Census. The participant reported on the health of their primary caregiver via a verbal autopsy (Becker et al., 2015), which were coded as whether their caregiver was sick with HIV/AIDS and whether their caregiver had a physical disability. Participants indicated whether they had experienced HIV-related stigma (via Stigma-by-Association Scale; Boyes et al., 2013) due to someone in their house being HIV-positive, whether they had a physical disability, whether they used alcohol or drugs for nonmedical purposes (via the Child Behavior Checklist; Achenbach, 1992; and via items from the National Survey of HIV and Risk Behavior Among Young South Africans; Reproductive Health Research Unit, 2005), and whether they had missed more than a consecutive week of school in the past year.

#### Social Protections

The participants’ receipt of South African government social protections was assessed, including whether their household received a monthly child grant (280 ZAR per month at the time of the interview), whether the participant was provided with free school meals, and whether the participant received free (no-fee) schooling.

### Analyses

Descriptive statistics (frequencies and percentages) for the measures of interest were calculated for the sample. Subsequently, LCA were carried out to determine the number of heterogeneous subgroups based on violence victimization experiences as well as demographic, household, and social protection measures. LCA allows for the observation of patterns of violence and other variables in participants’ lives as they appear in the data without requiring definition of specific patterns or grouping prior to analyses. Thus, the underlying subgroups are able to emerge from the models based on the patterns that exist among the sample, which allows for the observation of organic polyvictimization patterns beyond those strictly defined by preexisting measurement definitions or frameworks (Lasky et al., 2021). LCA models included all the variables listed above, which were all included as binary measures. Models were tested with class sizes ranging from a 1-class to an 8-class model (Lo et al., 2001; Nylund et al., 2007). Analyses were performed using MPlus version 8 (Muthén and Muthén, 1998-2017).

Preliminary models examined univariate entropy values to identify potential demographic, household, or social protection measures that were not meaningfully contributing the class assignments. Potential measures with abnormally low (<0.05) univariate entropy values were not included in the assessed models considered for the final model (see Table 2). Excluded measures included those for the participant’s age, whether they were orphaned, whether no one in the household was employed, and whether the adolescent was HIV-positive. All models presented, for which diagnostic criteria and fit indices are provided below, included the same set of measures for violence victimization, demographic, household, and social protection, as listed above. Final model selection of the optimal number of classes was determined using Bayesian information criterion (BIC), sample-size adjusted BIC, Akaike information criterion (AIC), Vuong–Lo–Mendell–Rubin likelihood ratio test (VLMR LRT) value, and entropy. Smallest class count and smallest class size percentage were also assessed, toward ensuring that class sizes were not smaller than 5% of the total sample (Weller et al., 2020). Lower values of BIC and AIC reflect a better fit of the current complex model (*k* classes) compared to the previous less complex model (*k*–1 classes), and significant *p*-values for VLMR LRT indicate that the model (*k*) has significantly improved fit compared to the previous less complex model (*k*–1) (Nylund-Gibson & Choi, 2018; Weller et al., 2020). Entropy values range from 0 to 1 with values indicating improved discriminability among classes with 1 representing perfect class separation; values above 0.8 are viewed as acceptable (Celeux & Soromenho, 1996; Ford et al., 2013; Weller et al., 2020). Model fit and the final class solution should be determined using the quantitative fit measures above, as well as the application of relevant theory or models to determine which solution produces classes that are distinct and meaningful (Nylund et al., 2007; Weller et al., 2020).

## Results

The eligible sample size included 3,401 participants. Table 1 presents the descriptive statistics for the sample, which included demographics of age, gender, and setting of their residence, as well as for violence, risk factors, and protective factors included in the LCA. The mean of the sample was 14.7 years with slightly more girls (56.4%) than boys and an even split between rural (49.8%) and urban residences. Violence victimization of any single form of violence ranged from 16.1% for sexual abuse to 66.6% for bullying. The sample experienced a high burden of poverty (hunger: 38.7%; necessities: 40.0%) and housing insecurity (20.6%) and many adolescents reported having an AIDS-sick caregiver (40.0%) or experiencing HIV-related stigma (43.3%). Nearly all participants received free school meals (89.5%), while approximately two-thirds received the child grant (63.0%) and free schooling (65.9%).

**Table 1.** Descriptive Statistics for Eligible Adolescent Participants (*n* = 3,401).

Participant characteristics	*n* (%)
Demographics	
Age (Wave 2) Mean (SD)	14.7 (2.2)
Gender	
Boy	1,482 (43.6)
Girl	1,919 (56.4)
Residence	
Rural	1,692 (49.8)
Urban	1,709 (50.2)
Violence	
Domestic violence exposure	1,472 (43.3)
Community violence	
Experienced	1,524 (44.8)
Witnessed	1,359 (40.0)
Physical abuse	2,009 (59.1)
Emotional abuse	1,781 (52.4)
Sexual abuse	549 (16.1)
Bullying	2,265 (66.6)
Risk factors	
Poverty	
Hunger	1,317 (38.7)
Necessities	1,360 (40.0)
Informal housing	701 (20.6)
Caregiver	
AIDS-sick caregiver	1,360 (40.0)
Caregiver disability	742 (21.8)
Disability	1,124 (33.1)
HIV-related stigma	1,472 (43.3)
School missed	824 (24.2)
Substance use	985 (29.0)
Protective factors	
Child grant	2,141 (63.0)
School meal	3,045 (89.5)
Free school	2,242 (65.9)

*Note.* *n* = count; *SD* = standard deviation.

The model fit indices are summarized in Table 2. Though the minimum AIC and BIC values occurred with the eight-class model, the VLMR LRT value was clearly nonsignificant (0.5177), indicating an eight-class model as a poor fit for the data. The next lowest set of AIC and BIC values occurred for the seven-class model, which did yield a significant VLMR LRT value (0.000) and for which the entropy was approximately similar to the other class models investigated with three to six classes. Additionally, the smallest class count and percentages were acceptable, lending confidence to the appropriateness of the seven-class model. Thus, the results for that model are presented here.

**Table 2.** Diagnostic Criteria and Model Fit Indices for the Latent Class Models for One to Eight Classes (Seven-Class Solution Optimal).

Number of Classes	VLMR LRT (*p* value)	Entropy	Small Class Count (n)	Smallest Class Size (%)	AIC	BIC	Adjusted BIC
1	—	—	3,401	100	83,625.193	83,747.830	83,684.281
2	0.000	0.732	1,215	35.7	79,973.128	80,224.533	80,094.257
3	0.000	0.814	852	25.1	77,948.251	78,328.424	78,131.421
4	0.000	0.833	694	20.4	76,497.110	77,006.051	76,742.322
5	0.000	0.810	593	17.4	75,932.526	76,570.236	76,239.780
6	0.000	0.806	279	8.2	75,725.581	76,294.059	75,896.875
**7**	**0.000**	**0.807**	**271**	**8.0**	**75,230.448**	**76,125.694**	**75,661.784**
8	0.5177	0.807	262	7.7	75,036.083	76,060.098	75,529.461

Adjusted BIC = Sample-size adjusted BIC; AIC = Akaike information criterion; BIC = Bayesian information criterion; VLMR LRT = Vuong–Lo–Mendell–Rubin likelihood ratio test.

Table 3 presents the results of the seven-class model, which produced two classes experiencing a high proportion of polyvictimization (Classes 1 and 2), four classes experiencing moderate polyvictimization (Classes 3, 4, 5, and 6), and one class with low polyvictimization experiences (Class 7). The exposure rates/likelihood proportion of experiencing each measure—the violence measures considered for polyvictimization, the risk factors, and the protective factors—are shown in Table 3, along with class size and entropy values. To help distinguish patterns of relatively low (<0.33), moderate (0.33–0.67), or high (>0.67) likelihoods of each violence or risk/protective measure within each class, the model values in Table 3 are shown in *italicized*, normal, and bold text to indicate the low, moderate, and high patterns, respectively, across the classes. In reading model results, the strongest contrasts are between the high (bold) and low (*italicized*) likelihoods and approximately similar likelihoods are those in the same likelihood category (Table 3: among high/bold, moderate/normal, and low/*italicized*) or adjacent likelihood categories. Classes were determined to reflect (a) high polyvictimization if they had three or more violence types classified as high likelihood and no more than two types as low likelihood, (b) moderate polyvictimization if four or five violence types were moderate or high likelihood (no more than three types with low likelihood), and (c) low polyvictimization if four or more types had low likelihood.

**Table 3.** Latent Class Likelihood Proportions for the Seven-Class Model for Polyvictimization, Risk Factors, and Protective Factors.

		Class Size (%)	Class 1	Class 2	Class 3	Class 4	Class 5	Class 6	Class 7	Univariate Entropy
		Polyvictimization Level^a^	486 (14)	611 (18)	323 (10)	473 (14)	271 (8)	623 (18)	614 (18)	
Category	Subcategory	Variable	High	High	Moderate	Moderate	Moderate	Moderate	Low	
Polyvictimization	Violence	Domestic Violence	0.474	0.417	0.505	0.406	0.600	0.575	*0.181*	0.039
		Community Violence (Experienced)	**0.762**	0.621	0.390	*0.214*	0.500	0.353	*0.304*	0.054
		Community Violence (Witnessed)	**0.715**	0.646	*0.156*	*0.057*	*0.182*	*0.155*	0.635	0.102
		Physical Abuse	**0.798**	**0.811**	0.617	0.534	**0.694**	0.563	*0.221*	0.064
		Emotional Abuse	**0.815**	**0.809**	0.486	0.334	0.664	0.425	*0.211*	0.076
		Sexual Abuse	*0.227*	*0.238*	*0.290*	*0.140*	*0.148*	*0.117*	*0.046*	0.030
		Bullying	**0.838**	**0.797**	**0.701**	0.477	**0.788**	**0.700**	0.437	0.045
Risk factors	Demographics	Rural	0.615	0.608	0.897	1	*0.052*	*0.015*	0.387	0.169
	Poverty	Hunger	**0.831**	0.645	0.332	*0.138*	*0.290*	*0.123*	*0.303*	0.091
		Necessities	**0.850**	0.658	*0.189*	*0.126*	*0.190*	*0.045*	0.554	0.121
	Household	Informal Housing	0.386	0.378	*0.006*	*0.005*	*0.035*	*0.052*	0.384	0.078
	Caregiver	AIDS-sick Caregiver	**0.930**	*0.163*	**0.768**	*0.163*	**0.815**	*0.130*	*0.260*	0.137
		Caregiver Disability	**0.835**	*0.012*	0.412	*0.011*	0.443	*0.003*	*0.085*	0.151
	Adolescent	Disability	**0.800**	*0.055*	**0.873**	*0.098*	**0.812**	*0.084*	*0.125*	0.163
		Stigma	**0.789**	0.540	0.602	*0.313*	0.459	*0.217*	*0.243*	0.062
		School Missed	0.468	0.472	*0.174*	*0.169*	*0.108*	*0.058*	*0.243*	0.052
		Substance Use	0.544	0.487	*0.068*	*0.040*	*0.171*	*0.093*	0.448	0.077
Protective factors	Grant	Child Grant	0.570	0.580	**0.807**	**0.742**	**0.677**	0.600	0.553	0.027
	Schooling	School Meal	**1**	**1**	**0.993**	**0.992**	**0.669**	**0.679**	**0.991**	0.076
		Free School	**0.813**	**0.852**	**0.974**	**0.992**	*0*	*0.080*	**0.806**	0.193

*Low likelihood* (<0.33); Moderate likelihood (0.33–0.67); High likelihood (>0.67).

a Classes were determined to reflect (a) high polyvictimization if they had three or more violence types classified as high likelihood and no more than two types as low likelihood, (b) moderate polyvictimization if four or five violence types were moderate or high likelihood (no more than three types with low likelihood), and (c) low polyvictimization if four or more types had low likelihood.

### Classes 1 and 2: High Polyvictimization

Classes 1 and 2 showed patterns of high polyvictimization. Both showed moderate to high likelihoods of domestic violence, community violence (experienced and witnessed), physical abuse, emotional abuse, and bullying. They also presented two of the three highest likelihoods (Class 1: 0.227; Class 2: 0.238) of experiencing sexual abuse across the seven classes. Both high polyvictimization classes were also characterized by high poverty (hunger: 0.645–0.831; necessities: 0.658–0.850), a high receipt of school-related protective factors (school meal: 1; free school: 0.813–0.852), a moderate receipt of the child grant (0.570–0.580), and a mix of adolescents living in rural and urban settings (rural: 0.608–0.615).

Class 1 was most distinguished from Class 2 by the caregiver AIDS and disability-related risk factors. Adolescents in Class 1 had a high likelihood of having an AIDS-sick caregiver (0.930), experiencing stigma due to HIV status of someone in their household (0.789), having a caregiver with a disability (0.835), and having a disability themselves (0.800). Thus, while Class 1 adolescents had approximately similar polyvictimization outcomes to those in Class 2, individuals in Class 1 were much more burdened by HIV/AIDS and disability than their Class 2 peers.

### Classes 3, 4, 5, and 6: Moderate Polyvictimization

Classes 3, 4, 5, and 6 showed patterns of moderate polyvictimization. In general, compared to Classes 1 and 2, participants in these four classes experienced a lower likelihood of each form of violence. However, these adolescents were not immune from encountering polyvictimization. All four classes have a moderate likelihood of being exposed to domestic violence (0.406–0.600) and a moderate to high likelihood of bullying victimization (0.477–0.788). Class 5 was marked by somewhat higher likelihoods of experiences of (0.500) and witnessed (0.182) community violence, physical abuse (0.694), and emotional abuse (0.664) compared to Classes 4, 5, and 6. However, Class 3 was set apart by the highest likelihood of sexual abuse (0.290) of all seven classes alongside the moderate likelihoods of other forms of violence in this class.

Classes 3 and 4 were both composed of participants from primarily rural (0.897–1) settings. They both showed a low to moderate likelihood for the measures of poverty (hunger: 0.138–0.332; necessities: 0.126–0.189) and a high likelihood of protective factors (child grant: 0.742–0.807; school meal: 0.992–0.993; free school: 0.974–0.992). Like Class 1 compared to Class 2, Class 3 was set apart from Class 4 by increased likelihood of caregiver and/or household HIV/AIDS and caregiver and/or adolescent disability impacting the adolescents’ lives. Compared to the low likelihood (0.011–0.313) in Class 4, Class 3 participants had a moderate likelihood of having a caregiver with a disability (0.412) and experiencing HIV-related stigma (0.602) and a high likelihood of having an AIDS-sick caregiver (0.768) and having a disability (0.873). Class 3 was thus notable because these adolescents from predominately rural areas experienced a relatively high likelihood of sexual abuse among a pattern of moderate polyvictimization while also being more heavily impacted by HIV/AIDS and disability than their rural peers in Class 4.

Classes 5 and 6 consisted of nearly exclusively urban participants (rural: 0.015–0.052) with a low likelihood of poverty (hunger: 0.123–0.290; necessities: 0.045–0.190). Though the two classes had approximately the same likelihood of receiving the child grant (0.600–0.677) and school meals (0.669–0.679), they had a nearly zero likelihood of attending school for free (0.000–0.080). Similar to the other classes paired by polyvictimization level and degree of rurality (Class 1 vs. Class 2; Class 3 vs. Class 4), participants in Class 6 had a low likelihood of experiencing HIV/AIDS or disability risk factors, while those in Class 5 had a moderate to high likelihood of having an AIDS-sick caregiver (0.815), a caregiver with a disability (0.443), a disability (0.812), or experienced HIV-related stigma (0.459).

### Class 7: Low Polyvictimization

Class 7 showed a pattern of low polyvictimization, which distinguished these participants from the rest of the sample. Though they lived in a mix of urban and rural settings (rural: 0.387), which were perhaps entangled in violence, given their moderate likelihood of witnessing community violence (0.635), these adolescents displayed a low likelihood of experiencing abuse (physical: 0.221; emotional: 0.211; sexual: 0.046), community violence (0.304), or being exposed to domestic violence (0.181). For all forms of violence, including bullying (0.437), with the exception of community violence, they had the lowest likelihood of all seven classes of experiencing any form of violence. Though they were more likely to experience poverty (hunger: 0.303; necessities: 0.554), live in informal housing (0.384), and use substances (0.448) compared to those in the moderate polyvictimization classes, they experienced relatively little of the violence that seemed to impact these peers. They had a low likelihood of having risk factors related to HIV/AIDS or disability (AIDS-sick caregiver: 0.260; caregiver disability: 0.850; disability: 0.125; HIV-related stigma: 0.243), and nearly matching likelihoods for protective factors as in Classes 1 and 2 (child grant: 0.553; school meal: 0.991; free school: 0.806).

## Discussion

This study sought to investigate the patterns of polyvictimization and relevant risk and protective factors that existed among sampled South African adolescents from low-resource, highly deprived settings. Using latent class analysis as a person-centered approach to explore the underlying subgroups for violence, household and individual risk factors, and government-provided protective factors in the sample, this study examined how polyvictimization varied among the adolescents in a contextually sensitive manner as classes accounted for patterns of poverty, HIV/AIDS-burden, disability, substance use, and receipt of government benefits that were found among these polyvictimization groupings. This work expanded work previously done in South Africa by including and highlighting the violence patterns in rural South African communities, meeting the need to examine violence exposures among adolescents from disadvantaged communities, and considering forms of violence beyond child abuse in these communities (Herrero Romero et al., 2021; Kaminer et al., 2013; Meinck et al., 2016). The results supported a seven-class model, which grouped participants into two high polyvictimization classes, four moderate polyvictimization classes, and one low polyvictimization class. The high polyvictimization classes have approximately similar patterns of violence victimization across the seven types of violence in that class members have high likelihood (>0.67) or a relatively high likelihood (relative to other classes, e.g., sexual abuse) of experiencing each form of violence. The moderate polyvictimization classes experienced a lower burden of violence victimization across the violence types, represented via a dampened likelihood of victimization compared to the high polyvictimization classes for all or nearly all forms of violence. The low polyvictimization class experienced a low likelihood of direct violence victimization experiences (<0.33) for nearly all forms of violence. The notable exception to this low or relatively low likelihood of violence experiences is the moderate likelihood of these class members witnessing community violence, that is, someone being shot or stabbed. This seems to demonstrate that despite these participants living in communities where violence is present and occurring, they are somehow able to avoid the other forms of victimization that are common among participants in the other six polyvictimization classes. As previous work examining polyvictimization in South Africa has emphasized, adolescents in this sample experienced a diversity of polyvictimization profiles and were not all similarly exposed to violence (Herrero Romero et al., 2019). Consideration of the context—at the individual, household/family, and community level—of the violence experienced deepens our understanding of these patterns produced leading to a more holistic, contextually sensitive understanding of polyvictimization in this sample. This holistic view builds on and responds to calls for explorations of polyvictimization that incorporate context and move beyond only examining violence groupings (Butcher et al., 2016; Davis et al., 2019).

The factors considering the context of these polyvictimization classes—the patterns of risk and protective factors–provided interesting insights. The high and moderate polyvictimization classes contrasted in their setting (rural vs. urban) and burden of certain risk factors. The high polyvictimization classes were composed of a similar proportion of participants from rural and urban settings, demonstrating that the heavy burden of polyvictimization could occur across setting or community type. By contrast, the moderate polyvictimization classes were separated based on setting, with two urban classes and two rural classes. These findings support previous work with a sample of adolescents from Cape Town, which found that context matters for South African adolescents, as neighborhood and other contextual factors impacted their violence exposures and polyvictimization experiences (Kaminer et al., 2013).

Within the high polyvictimization classes, one group was marked by a notably higher burden of poverty, household disability (caregiver and adolescent), and HIV/AIDS than their peers with similar polyvictimization patterns. Likewise, with each of the rural–urban class pairs in the moderate polyvictimization classes, one group experienced this same pattern of a greater burden of poverty, disability, and HIV/AIDS. Though they experienced a relatively moderate poverty and substance use burden, the low polyvictimization class was not greatly burdened by disability or HIV/AIDS.

Receipt of government-provided benefits (child grant; school meal; free school) was relatively high across all the seven classes and the levels of polyvictimization. The exception to this was the low receipt of free schooling among participants from primarily urban settings. The receipt of these social benefits did not appear to be protective against any level of polyvictimization, as the pattern for the receipt of these benefits was relatively uniform across all seven classes. For example, participants in the high and low polyvictimization classes had nearly the same likelihood of receiving all three of the social protections.

These results indicate the power of considering violence using a polyvictimization framework when coupled with employing a person-centered method such as latent class analysis. If this study’s research questions had sought to understand the connection of violence to other factors in adolescents’ lives when considering only one form of violence, or only examining the three common forms of child abuse, many of the nuances presented here would have been missed. Building on the work of Turner et al. (2016) and Lasky et al. (2021) who showed the diversity of violence events using latent class analysis, these findings demonstrate what has been found in other settings: experiences of violence in the lives of adolescents are often not isolated to a single form of violence. Instead, many adolescents experience multiple forms of violence (Butcher et al., 2016; Finkelhor et al., 2011; Herrero Romero et al., 2021), and, in the case of our study, multiple patterns of polyvictimization emerge with their different concomitants. Research among additional impoverished settings in Africa has likewise found that children experiencing multiple forms of violence are also subject to additional burdens of adversity and stressors (Ismayilova et al., 2016). Given that adolescents who experience polyvictimization seem to have worse outcomes stemming from their violence exposures than those who are not polyvictimized, consideration of adolescent violence though a polyvictimization lens seems crucial to understand the complexity of violence in adolescents’ lives (Adams et al., 2016; Butcher et al., 2016; Song et al., 2022).

Our findings also build on and connect to work that has previously been done to examine the nature of abuse and violence, poverty, and HIV/AIDS in low-resource South African contexts. As in our sample, previous examinations of polyvictimization among South African adolescents found the experiences to be common and occurring across multiple settings (home, community, school; Kaminer et al., 2013) and to be influenced by individual, proximal (household), and distal (urban/rural context) factors with intersecting factors resulting in increased associations with polyvictimization (Leoschut & Kafaar, 2017). Work by Steinert et al. (2017) identified links between AIDS and poverty, while Meinck, Cluver, Orkin et al. (2017) demonstrated that adolescents impacted by HIV/AIDS and poverty faced a higher risk of abuse, and adolescents from AIDS-ill families experienced increased abuse risk mediated by poverty and disability (Meinck et al., 2015a).

Given the connection between violence and antiretroviral nonadherence found among HIV-positive adolescents in South Africa (Cluver et al., 2018, 2021, 2022), these polyvictimization results further illuminate the complex relationship between HIV/AIDS and violence in low-resource settings, as polyvictimization levels were associated with the HIV status of household members rather than the adolescent’s status. Adolescents in the high polyvictimization groups were most likely to have missed significant time at school, which is similar to work that demonstrated a connection between poly-violence and educational delays in South Africa (Herrero Romero et al., 2019, 2021). Likewise, community violence was identified as a predictor of sexual abuse for South African adolescent girls, findings which fit well into the polyvictimization framework with earlier events of adolescent violence linking to subsequent violence taking different forms (Meinck et al., 2015b). Overall, these latent class results support previous studies specific to South Africa and expand the scope to include explicit examinations of polyvictimization alongside potential risk and protective factors for violence, allowing us to better understand the contexts in which these adolescents live.

### Limitations

The participants sampled for this study lived in two regions in South Africa, and data were collected over a decade ago, meaning that the results are not generalizable beyond those settings and timeframe. However, random sampling methods and low refusal rates among eligible participants mean that the sample is likely representative of adolescents from those regions in 2010 to 2012. Additionally, the sampled regions were selected to capture the highly deprived settings in which many South African adolescents grew up, so the results may be applicable to other similar low-resource settings in South Africa or southern African contexts. The seven measures of violence included in the analysis represent all forms of violence about which participants were surveyed. However, other forms of violence victimization relevant to adolescents were not included in the data including dating/partner violence, labor or sexual exploitation, and sibling violence. When considering the relationships between multiple forms of violence via polyvictimization, this paper is limited by a weakness of the overall polyvictimization field, which lacks a cohesive framework or theory to explain the interactive effects or patterns of violence within polyvictimization, though this work is improved via its use of a person-centered method to tease out heterogeneous violence groupings within the sample (Emery et al., 2023).

The age of the data is a noted limitation; however, this sample represents valuable data for this setting and population; more recent data in South Africa come from a birth cohort that draws from an urban sample, while this sample captures data from rural communities as well (Richter et al., 2018). The age of the data also represents an era in South Africa when government-provided social supports were weaker. For example, at the time of sampling, the child grant was in the process of being implemented, so not all families in the sample who were eligible for the grant were receiving it. Additionally, the child grant eligibility only extended up to age 14 at the time, though eligibility now extends until the child is age 18. Thus, the current social supports in South Africa may better protect against polyvictimization than our findings show though we are unable to conclude that from these results. Conversely, the cumulative burden of multiple crises over the past few years—COVID-19 pandemic, energy crisis, rising cost of living—may have exacerbated some of the strain on South African households beyond what is detected in this older data; as a result, these findings may underreport the current nature of polyvictimization in these settings.

The diversity of the participants in the sample and constraints of the data should be acknowledged. The data drew from two settings in South Africa representing multiple South African heritages, and all participants were Black adolescents with a non-English home language. However, these settings were selected to recruit participants often overlooked in samples from communities with greater resources or more racial/ethnic or economic diversity, resulting in a sample of participants who are demographically similar in some ways but who add to the diversity of samples from South Africa. The gender diversity in the sample is limited to girls and boys, as identities outside of the gender binary were not asked when the data were collected. The sampled participants come from populations who were historically marginalized and deprived under South African apartheid, which was lifted just as or before these sampled adolescents were born. Thus, their inclusion represents a uniquely diverse sample who have primarily been raised in a post-Apartheid South African but who continue to feel the historical inequities of the previous oppression.

### Implications

These findings highlight the diverse polyvictimization and additional burdens experienced by South African adolescents and have several implications for future research, policy, and practice. A strength of LCA is the enriched understanding of the characteristics and sizes of each class produced, as the features and relative size of each class are essential for research, policy, and implementation to shape an understanding of how prevalent each class experience is in the sample. Future research should examine the physical and mental health effects felt by South African adolescents by levels of polyvictimization, as well as explore what causal links exist between the risk factors and polyvictimization, including parenting behaviors and resource access. Investigating patterns of violence and polyvictimization across developmental stages, including young childhood, would also be a valuable addition to improve understanding of when first violence exposures and incidents of revictimization occur within polyvictimization trajectories (Ferrajão et al., 2022).

Policy and programming responses are needed to implement and examine a wider range of social benefits or programming that may prevent polyvictimization, as well as better protecting the most vulnerable in society from violence, poverty, disability, and the detrimental social and health effects of HIV/AIDS. These findings highlight the challenges of violence and other burdens by rural communities and require policy and programming responses to consider the distribution of resources to rural populations. Responses should include more extensive screening in healthcare settings for factors related to polyvictimization to allow providers to uncover and provide required interventions for polyvictimization patterns and elements of violence vulnerability (e.g., poverty, HIV/AIDS, and disability) in the lives of adolescents. Relatedly, targeted interventions are needed to address a broad scope of adolescent violence experiences. This requires a cohesive approach and integrated strategies to prevent and intervene in violent events in multiple settings, rather than prioritizing a single form of adolescent violence, and efforts to integrate policy and legal responses to prevent violence with prevention programming across multiple settings (families, schools, and communities), as has been emphasized elsewhere (Miedema et al., 2023). Currently, South Africa does not have a cohesive response and approach to addressing all forms of violence experienced by children. Efforts to implement parenting programs in South African communities are valuable but only address issues of child abuse perpetrated by caregivers. There is a need to develop an overarching agenda in South Africa and similar settings to develop policy and practice that target the vast scope of childhood violence experiences in an integrated manner. Whereas South African policy acknowledges and mandates reporting for harms beyond child abuse and neglect (e.g., bullying requires a mandated report; Harrington-Johnson, 2023), the practice response fails to prevent the vast scope of violence experienced by children or meet the needs of those exposed to or experiencing a diversity of violence. Previous work to understand accelerators for violence prevention in South Africa may provide a valuable framework throughout which to develop an approach that considers and influences violence across multiple types and settings (Cluver et al., 2020).

Given the interconnected nature and correlation between forms of adolescent violence, all violence programming and screening should be designed to probe for and respond to polyvictimization once one form of violence has been detected or disclosed; and diminution of crucial risks associated with polyvictimization, such as HIV/AIDS, poverty, and disability, should be included in the program and policy response to adolescent violence. All evaluation work in these settings should include multiple measures of violence to capture the diversity of experiences in the lives of children and work within a theory of change that considers the links and overlaps between forms of childhood violence. These responses should be coupled to future research studying the causal prevention mechanisms or interrupters of polyvictimization. Together, any prevention or intervention program or policy targeting or related to adolescent violence must consider the diversity of violence experienced by adolescents; to continue to design program or policy with a single or limited number of forms of violence as a priority outcome is to miss the complex and interconnected web of violence that many adolescents experience.

## Conclusion

This analysis highlighted the patterns of violence alongside risk and protective factors among South African adolescents living in highly deprived settings. The results provided a contextually sensitive understanding of polyvictimization in these communities, demonstrating how experiences of differing levels of polyvictimization (high, moderate, low) occurred alongside burdens of HIV/AIDS, disability, and poverty and within urban and rural settings. To consider polyvictimization through a contextually sensitive lens seems essential, as violence—particularly experiences of multiple forms of violence—does not happen within a vacuum absent of an adolescent’s personal and community context. Additional research, practice, and policy must seek to prevent and intervene in the violence experienced by children in communities across South Africa while also addressing the drivers of violence and additional burdens faced by adolescents in order to holistically respond to their needs while adequately protecting their rights and health.

## Supplemental Material

sj-docx-1-jiv-10.1177_08862605241233273 – Supplemental material for Toward a Contextually Sensitive Understanding of Polyvictimization: A Latent Class Analysis of Violence, Risks, and Protections Among South African Adolescents From Highly Deprived Settings Supplemental material, sj-docx-1-jiv-10.1177_08862605241233273 for Toward a Contextually Sensitive Understanding of Polyvictimization: A Latent Class Analysis of Violence, Risks, and Protections Among South African Adolescents From Highly Deprived Settings by Hannabeth Franchino-Olsen, Mark Orkin and Franziska Meinck in Journal of Interpersonal Violence
