# Supplementary material for: Toward a Contextually Sensitive Understanding of Polyvictimization: A Latent Class Analysis of Violence, Risks, and Protections Among South African Adolescents From Highly Deprived Settings
Source: J Interpers Violence. 2024 Feb 26;39(15-16):3591–618. doi: 10.1177/08862605241233273 (PMC11283737; doi:10.1177/08862605241233273)
Supplement: sj-docx-1-jiv-10.1177_08862605241233273 – Supplemental material for Toward a Contextually Sensitive Understanding of Polyvictimization: A Latent Class Analysis of Violence, Risks, and Protections Among South African Adolescents From Highly Deprived Settings [file sj-docx-1-jiv-10.1177_08862605241233273.docx]

**Appendix 1: Information for included Young Carers phase 1 and phase 2 measures**

|  | **Young Carers, phase 1 (2010–11) and phase 2 (2011–12)** |
| --- | --- |
|  | **DEMOGRAPHIC INFORMATION** |
| Age, gender, residence | Child age, gender and basic demographic information (including urban/rural residence) were asked using items from the South African Census 2001 (Statistics South Africa, 2003). |
|  | **VIOLENCE** |
| Violence & Abuse | Exposure to family conflict and domestic violence was measured using items from the UNICEF Measures for National-level monitoring of orphans and other vulnerable children (Snider & Dawes, 2006) (2 items). Physical abuse (2 items) and emotional abuse (10 items) were measured using items from the UNICEF Measures for National-level Monitoring of Orphans and Other Vulnerable Children (Snider & Dawes, 2006). Exposure to sexual abuse was measured using three items from the Juvenile Victimization Questionnaire (JVQ) (Finkelhor et al., 2005) and used in our previous studies in South Africa. Access to help and reactions to disclosure of abuse were measured using three items from the ISPCAN International Child Abuse Screening Tool (Zolotor et al., 2009). Sense of safety in the home was measured using items from the National Primary Schools Violence Survey 2007 (Burton, 2008) (4 items). A further item assesses intra-household discrimination (i.e. differential allocation of food between fostered and biological children) and was developed in qualitative pilot work (1 item). |
| Community Violence | Community-level trauma was measured using items from the Child Exposure to Community Violence (CECV) Checklist (Richters & Martinez, 2004), adapted to reflect commonest community traumas in South Africa, as identified by national police statistics (South African Police & Management, 2005) |
| Bullying | Bullying was measured with the 9-item, standardised ‘Social and Health Assessment Peer Victimization Scale’ (Ruchkin et al., 2004), used in research with vulnerable children in Cape Town (Ward et al., 2007) and in our previous study of AIDS-orphanhood. This scale was adapted from the Multidimensional Peer Victimization Scale, and showed α=.82 in a US validation study (Mynard & Joseph, 2000) and α=.85 in our AIDS-orphanhood study . Items include being called names, being hit or threatened and having possessions broken or stolen. This measure generates a total global score of exposure to bullying. |
|  | **RISK FACTORS** |
| Housing & Necessitates | Poverty was measured using a range of tools. Items from the South African Census (Stats SA) determined whether children lived in formal, informal (i.e. shacks) or traditional structures. Poverty was also measured by measuring access to the top 8 socially-perceived necessities for children, as identified by the Centre for South African Social Policy in the ‘Indicators of poverty and social exclusion project’ (Barnes & Wright, 2012), and endorsed by over 80% of the South African population in a nationally-representative survey (the South African Social Attitudes Survey 2006) (Pillay et al., 2006). These include items such as ‘enough clothes to keep you warm and dry’ and ‘3 meals a day’. |
| Hunger | Food insecurity was measured using 2 items from the South African National Food Consumption Survey (1999) (Labadarios et al., 2003). |
| Caregiver: AIDS-sick caregiver or Caregiver disability | Parental morbidity was measured at two separate stages. Firstly, in the ‘Road of Life’ (an adaptation of a social work tool ‘the river of life’ (Buchanan, 2002) children identified where parental sickness caused a change in living environment, a household move, or was a significant milestone in their lives. Secondly, children identified on the Household map whether there were any sick or disabled people in the home. Where there was a sick person, the children identified who they helped look after most, and completed a ‘confidential sickness report sheet’. This sheet was placed in a separate, sealed envelope (a technique used for sensitive information in the Cape Area Panel Survey and recommended by our Child Advisory Team). The sickness report sheet identified chronicity of illness, extent of illness and frequency of illness, using items from the World Health Organisation International Classification of Functioning, Disability and Health (World Health Organization, 2003) (4 items). Subscales from the ‘Activity limitation and participation restriction’ section (mobility and self-care domains) were used to assess extent of disability (7 items). Cause of sickness was identified using a verbal autopsy questionnaire (Lopman et al., 2006), developed for use in areas with over 20% HIV-prevalence, and showing sensitivity of 83% and specificity of 75% in a Zimbabwean cohort. This questionnaire was designed primarily to identify symptoms of AIDS, and in order to a) reduce the potentially stigmatizing nature of this and b) identify other types of illness affecting caregivers, added items include the most common causes of adult illness in South Africa, as identified in the Demographic and Health Survey (2003) and the Health Systems Trust Annual Review (2006) (Department of Health & Medical Research Council, 2007; Ijumba et al., 2006) (18 items). The verbal autopsy has been shown to reliably match children’s reports of caregiver’s HIV status with the adults’ reports (Becker et al., 2015). |
| Disability | Two items measuring physical disability included visual disability and others. |
| Stigma | Two scales were used to assess exposure to AIDS-related stigma. Four items were developed from the previous study of AIDS-orphanhood by stigma researchers in South Africa and from qualitative data, and showed α = .90. In addition, a recently-developed ‘stigma-by-association scale’ has been validated to measure perceived stigma amongst US adolescents with HIV+ mothers (Mason et al., 2010). This scale was adapted and reduced and validated in South Africa (Boyes et al., 2013). |
| Education & School Attendance | Education items were developed with the South African Department of Education. Some items from the ‘Young Lives’ study (Boyden & Dercon, 2008) were used to determine some education outcomes, others were identified by NACCA and NGOs. Items included: age of school enrolment, grade for age, repetition of grades and reasons for repetition, failure of grades and reasons for failure, school dropout and reasons. We measured number of different schools attended, and migration within and between provinces. We also examined ability to pay school fees. We measured how frequently they missed more than a week of school in the past year and the reasons for the absences, as well as which days they missed in the past week and the reasons. For young carers, we measured daily allocation of time for school and homework. Items also examined missing school due to responsibilities. Further items examined whether children felt that their teacher understood their home situation, and whether children have accessed counseling at school. |
| Substance Use: [Alcohol or Drug Use](https://apps.who.int/iris/bitstream/handle/10665/67205/WHO_MSD_MSB_01.6a.pdf?sequence=1) | One item from Child Behaviour Checklist (Achenbach, 1992) delinquency subscale addresses drug and alcohol use. National survey of HIV and risk behaviour amongst young South Africans (Reproductive Health Research Unit, 2005) (4 items) measuring multiple types of substance use. |
|  | **PROTECTIVE FACTORS** |
| Government Grants & Schooling | Receipt within the household of the major forms of *social security transfers* (Child Support Grant, Foster Care Grant, Pension, Disability Grant and Care Dependency Grant) was measured, and reasons for *non-receipt of grants* assessed using the most-identified causes of non-receipt in a study of welfare access in South Africa (Noble et al., 2007) (5 items). Respondents identified whether their *school* was a no-fees school, or whether they had a fees exemption (3 items). They also identified whether they had state-provided school uniform, school transport, free school text books or school feeding scheme (items identified by the Department of Social Development). (3 items). |

**References**

Achenbach, T. (1992). *Manual for the Child Behaviour Checklist/2-3 and 1992 Profile*. University of Vermont.

Barnes, H., & Wright, G. (2012). Defining child poverty in South Africa using the socially perceived necessities approach’. In A. Minujin & S. Nandy (Eds.), *Global Child Poverty and Well-Being: Measurement, Concepts, Policy and Action* (pp. 135–154). Policy Press.

Becker, E., Kuo, C., Operario, D., Moshabela, M., & Cluver, L. (2015). Measuring child awareness for adult symptomatic HIV using a verbal assessment tool: Concordance between adult–child dyads on adult HIV-associated symptoms and illnesses. Sexually Transmitted Infections, 91(7), 528–533. https://doi.org/10.1136/sextrans-2014-051728

Boyden, J., & Dercon, S. (2008). Young Lives: An international study of childhood poverty. In *Www.younglives.org.uk*. www.younglives.org.uk.

Boyes, M., Mason, S., & Cluver, L. (2013). Validation of a brief stigma-by-association scale for use with HIV/AIDS-affected youth in South Africa. *AIDS Care*, *25*, 215–222. https://doi.org/10.1080/09540121.2012.699668

Buchanan, A. (2002). *Social work tools for assessment of children: Vol. Lecture*. Oxford University.

Burton, P. (2008). *National Primary School Violence Survey 2007*. Centre for Justice and Crime Prevention.

Department of Health, & Medical Research Council. (2007). *Demographic and Health Survey 2003*. Department of Health.

Finkelhor, D., Hamby, S. L., Ormrod, R., & Turner, H. (2005). The Juvenile Victimization Questionnaire: Reliability, validity, and national norms. *Child Abuse & Neglect*, *29*, 383–412. https://doi.org/10.1016/j.chiabu.2004.11.001

Ijumba, P., Padarath, A., & Health Systems Trust. (2006). *South African Health Review 2006: Vol. Durban*. Health Systems Trust.

Labadarios, D., Maunder, E., Steyn, N., MacIntyre, U., Swart, R., Gericke, G., Nesamvuni, E., Huskisson, J., Vorster, H., & Dannhauser, A. (2003). National food consumption survey in children aged 1-9 years: South Africa 1999. *Forum Nutr*, *56*, 106–109.

Lopman, B., Barnabas, R., Boerma, T., Chawira, J., Gaitskell, K., Harrop, T., Mason, P., Donnelly, C., Garnett, G., Nyamukapa, C., Gregson, S., Boerma, J., & Chawira, G. (2006). Creating and validating an algorithm to measure AIDS mortality in the adult population using verbal autopsy. *Public Library of Science Medicine*, *3*, e312.

Mason, S., Berger, B., Ferrans, C., Sultzman, V., & Fendrich, M. (2010). Developing a Measure of Stigma by Association With African American Adolescents Whose Mothers Have HIV. *Research on Social Work Practice*, *20*, 65–73. https://doi.org/10.1177/1049731508330223

Mynard, H., & Joseph, S. (2000). Development of the Multidimensional Peer-Victimization Scale. *Aggressive Behavior*, *26*.

Noble, M., Wright, G., & Cluver, L. (2007). Conceptualising, defining and measuring child poverty in South Africa: An argument for a multidimensional approach. In A. Dawes, R. Bray, & A. Van der Merwe (Eds.), *Monitoring child rights and wellbeing. A South African Approach*. HSRC Press.

Pillay, U., Roberts, B., & Rule, S. (2006). *South African Social Attitudes. Changing Times, Diverse Voices* (Vol. 1). HSRC Press.

Reproductive Health Research Unit. (2005). *HIV and sexual behaviour among young South Africans: A national survey of 15-24 year olds*. loveLife.

Richters, J., & Martinez, P. (2004). Violent Communities, family choices and children’s chances: An algorithm for improving the odds. *Development and Psychopathology*, *5*, 609–627.

Ruchkin, V., Vermeiren, R., & Schwab-Stone, M. (2004). *The Social and Health Assessment (SAHA): Psychometric developmental summary*. Yale University.

Snider, L., & Dawes, A. (2006). *Psychosocial Vulnerability and Resilience Measures For National-Level Monitoring of Orphans and Other Vulnerable Children: Recommendations for Revision of the UNICEF Psychological Indicator*. UNICEF.

South African Police, S., & Management, S. S. (2005). *Annual Report of the South African Police Service 2004/2005*. SAPS.

Statistics South Africa. (2003). *Census 2001: Census Database*.

Ward, C. L., Martin, E., Theron, C., & Distiller, G. B. (2007). Factors affecting resilience in children exposed to violence. *South African Journal of Psychology*, *37*, 164–187.

World Health Organization. (2003). *ICF Checklist Version 2.1a, Clinician Form for International Classification of Functioning, Disability and Health*. WHO.

Zolotor, A. J., Runyan, D. K., Dunne, M. P., Jain, D., Péturs, H. R., Ramirez, C., Volkova, E., Deb, S., Lidchi, V., Muhammad, T., & Isaeva, O. (2009). ISPCAN Child Abuse Screening Tool Children’s Version (ICAST-C): Instrument development and multi-national pilot testing. *Child Abuse & Neglect*, *33*, 833–841. http://dx.doi.org/10.1016/j.chiabu.2009.09.004
